# Effect of a Papain-based Chemomechanical Agent on Structure of Dentin and Bond Strength: An *in vitro* Study

**DOI:** 10.5005/jp-journals-10005-1504

**Published:** 2018-06-01

**Authors:** Sruthi Nair, Roopa R Nadig, Veena S Pai, Yashwanth Gowda

**Affiliations:** 1 Postgraduate Student, Department of Conservative Dentistry and Endodontics Dayananda Sagar College of Dental Sciences, Bengaluru Karnataka, India; 2 Professor and Head, Department of Conservative Dentistry and Endodontics Dayananda Sagar College of Dental Sciences, Bengaluru Karnataka, India; 3 Reader, Department of Conservative Dentistry and Endodontics Dayananda Sagar College of Dental Sciences, Bengaluru Karnataka, India; 4 Senior Lecturer, Department of Conservative Dentistry and Endodontics Dayananda Sagar College of Dental Sciences, Bengaluru Karnataka, India

**Keywords:** Carie-care, Carisolv, Energy-dispersive X-ray analysis, Papacarie.

## Abstract

**Aim:**

To evaluate the microtensile bond strength of teeth restored with packable composite after removing caries with three chemomechanical caries removal agents (Carisolv, Papacarie and Carie-care) and also to analyze its effect on chemical composition of dentin using energy-dispersive X-ray (EDX) analysis.

**Design:**

A total of 40 carious molars with 1 to 1.5 mm of remaining dentin were selected and divided into four groups of 20 each. Group I (control)—bur, group II—Carisolv, group III—Papacarie, group IV—Carie-care. A total of 15 samples from each group were restored with composite; 1 mm thick sections were made and were debonded under tensile load. Remaining five samples from each group were subjected to EDX for elemental analysis of dentin surface.

**Results:**

No statistically significant difference in the bond strength values and Ca/P ratio was observed between control group and three chemomechanical agents tested in the study.

**Conclusion:**

None of the chemomechanical agents tested in the study adversely affected the bond strength of composite resin to caries-affected dentin. Therefore, newer papain-based chemomechanical agent Carie-care can be considered as an equally effective economical alternative to commonly used agents Carisolv and Papacarie.

**How to cite this article:** Nair S, Nadig RR, Pai VS, Gowda Y. Effect of a Papain-based Chemomechanical Agent on Structure of Dentin and Bond Strength: An *in vitro* Study. Int J Clin Pediatr Dent 2018;11(3):161-166.

## INTRODUCTION

Introduction of adhesive restorative materials coupled with increased knowledge on the pathology of caries has brought about considerable change in the concepts of restorative dentistry allowing minimally invasive procedures. The best way to ensure maximum lifespan of the natural tooth is to respect sound tissues and protect them from damage.^1,2^

The conventional method of caries removal technique, although is quick and efficient, may result in unnecessary removal of the healthy and even the affected dentin that shows ability to remineralize. It may also be associated with pain and patient discomfort, necessitating administration of local anesthesia.^3-5^ To address these limitations, minimally invasive complementary caries removal methods were proposed like chemomechanical techniques, air abrasion with aluminum oxide or glass particle, sonoabrasion, ultrasonic instrumentation, and lasers. The common feature of these techniques is the selective removal of caries-infected tissue, while leaving intact the caries-affected tissue.^6^

The idea of chemomechanical caries removal was developed in the 1970s by an endodontist M Goldman using 5% sodium hypochlorite (5% NaOCl) in removing organic materials in the root canals. Among the chemo-mechanical agents, the most popular and gold standard is Carisolv system (MediTeam, Sweden). It consists of two syringes, one containing carboxymethylcellulose-based gel and amino acids (glutamine, leucine, and lysine); the other containing 0.5% NaOCl. Because of its disadvantages like time consumption, unpleasant smell, and taste, later in 2003, a Brazilian formulation for chemomechani-cal caries removal was introduced into the market under the trade name Papacarie (Formula e Acao, Sao Paulo, SP, Brazil) which is based on papain.^7-9^

Even though chemomechanical caries removal agents are useful in deep-seated caries lesions and pediatric patients, its use in developing countries is limited, because of the high cost of these materials. So recently with the idea of developing a cost-effective material, a newer product Carie-care (Unibiotech Pharmaceuticals, India) based on papain has been introduced into the market. Carie-care consists of papaya extract, clove oil, colored gel, chloramines, sodium chloride, and sodium methylparaben. Papain, a papaya extract, is a proteolytic enzyme similar to human pepsin and has antibacterial and anti-inflammatory properties. The absence of alpha-1-antitrypsin in infected tissues allows papain to break the partially degraded collagen and facilitates caries removal along with increased microbial properties. Chloramines help in the healing process, shorten tissue repair time, and have the potential of dissolving carious dentin by means of chlorination of partially degraded collagen. Clove oil has an analgesic and antiseptic action, whereas sodium methylparaben is used as a preservative. Studies have shown that the caries removing efficacy of Carie-care is equivalent to that of Papacarie and Carisolv.^9-11^

In the operative treatment of carious lesions in dentin, the morphology and nature of dentin surface treated with chemomechanical agents influence bonding of adhesive restorative material.^6^ Many studies have been conducted on Carisolv and Papacarie regarding the chemical and morphological analysis of residual dentin surface as well as on the bonding to caries-affected dentin.^12^ Previous studies have shown that bond strength to these irregular dentin surfaces treated with the above chemome-chanical caries removal agents is predominantly higher compared with conventionally prepared surfaces, and it was speculated that the roughened surface created by chemomechanical caries removal has better prerequisites for micromechanical retention and resin penetration.^13^ However, there are no studies evaluating the effect of new product Carie-care on adhesion of composite resin and also on dentin surface characteristics.

### Aim of the Study

The aim of the study is to evaluate the microtensile bond strength of teeth restored with packable composite after removing caries with three chemomechanical caries removal agents (Carisolv, Papacarie, and Carie-care) and also to analyze its effect on the chemical composition of dentin using EDX analysis and compare it with traditional technique of using bur to remove caries.

**Fig. 1:**
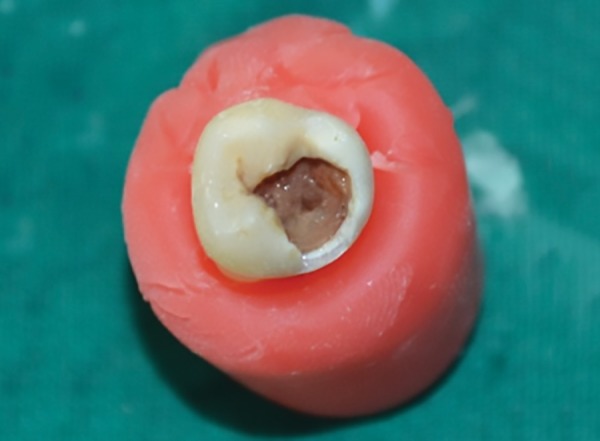
Extracted molars with coronal caries

## MATERIALS AND METHODS

Forty periodontally compromised freshly extracted molars with coronal caries with 1 to 1.5 mm of remaining dentin, with no indication of pulp exposure, was used in the study (Fig. 1). The carious teeth were sectioned through the deepest part of the occlusal fissure, perpendicular to the long axis of the crown to expose the carious lesion. The teeth were longitudinally sectioned with the diamond disk using a low-speed handpiece into two halves.

Twenty specimens were assigned for each group

- Group I: Carisolv excavation group, the carious lesion was treated with Carisolv™ (multimix gel; Medi Team, Sweden)
- Group II: Treated with Papacarie (Formula e Acao, Sao Paulo, Brazil)
- Group III: Treated with Carie-care (Unibiotech Pharmaceuticals, India)
- Group IV: Control group in which a tungsten carbide bur in an air turbine handpiece was used to excavate caries-infected dentin.

**Fig. 2:**
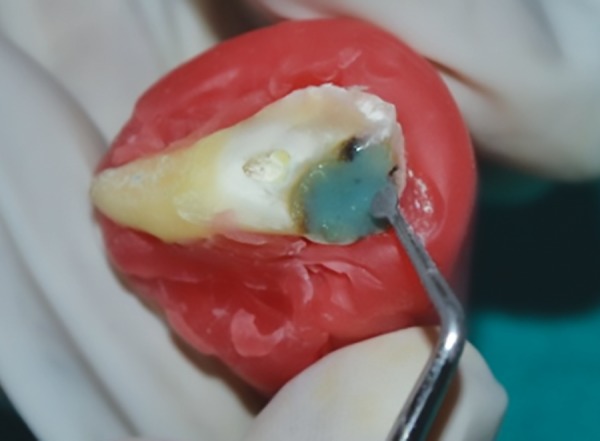
Excavating the caries after placing chemomechanical caries removal agent using a spoon excavator

**Fig. 3:**
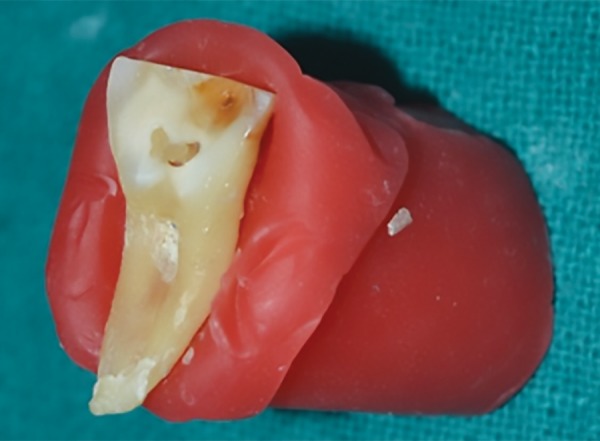
Caries-affected dentin left after caries excavation

All the above agents were used as per manufacturer’s instructions. Chemomechanical caries removing procedure for all the groups tested (groups I-III) were similar. The gel was left for 30 seconds prior to excavating the caries dentin using a spoon excavator (Fig. 2). Once the gel became cloudy, it was rinsed off with distilled water and the process was repeated until successive application of the gel failed to become cloudy. Figure 3 shows carious affected dentin left after carious excavation

### Microtensile Bond Strength

Fifteen samples from each group were built up using three layers of a posterior resin-based composite (Filtek Z350, 3M) to a height of approximately 5 mm using total etch adhesive system (single bond, 3M). Specimens were then stored in water at 37°C for 24 hours. The teeth were then individually fixed to a sectioning block using acrylic resin. Using hard tissue microtome 1 mm thick sections containing bonded caries-affected dentin specimens were obtained from each tooth depending on the size of caries lesion. The slices were then trimmed and shaped to form a gentle curve along the adhesive interface from both sides using superfine diamond burs to obtain a rectangular cross-sectional shape with the surface area of approximately 1 mm^2^. The beams were then attached to a custom-made jig and were attached to the Instron universal testing machine. A tensile load was applied at a cross-head speed of 0.5 mm/min until the beam fractured, and the load at which the fracture occurred was recorded.

### Energy-dispersive Analysis

Remaining five specimens from each group were subjected to EDX analysis to find out the chemical composition of the dentin. The cut surfaces were polished with 2000-grit silicon carbide paper and finished with diamond paste down to a 0.25-um particle size to the same depth as the excavated caries lesion. The smooth surface was subjected to elemental analysis under high vacuum at 1500x magnification using energy-dispersive system. The recorded values represented the average mineral weight percentages.

## RESULTS

The microtensile bond strength values (MPa) were calculated by dividing the force at failure by the cross-sectional area. One-way analysis of variance is used for statistical analysis.

**Graph 1:**
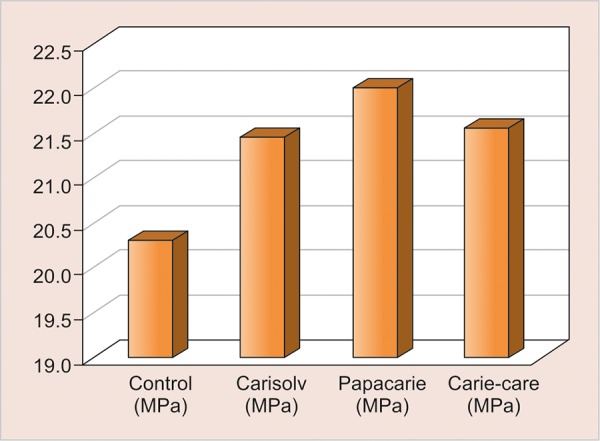
Mean microtensile bond strength values of four groups tested

The mean microtensile values of various groups are indicated in Table 1 and Graph 1. There was no statistically significant difference between the microtensile bond strength of four groups tested in the study.

**Graph 2:**
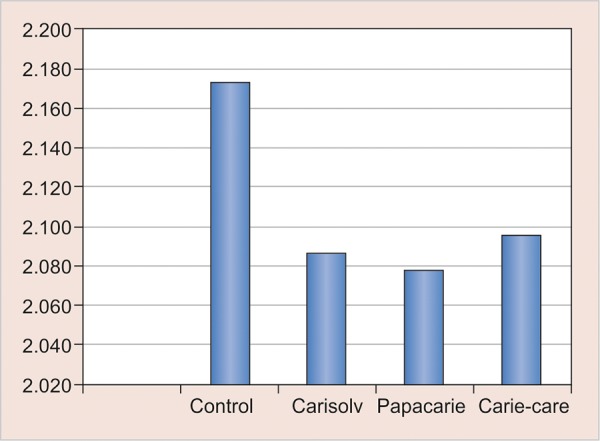
Ca/P ratio of all the four groups

There was no difference in the Ca/P ratio of all the groups tested in the study as shown in Table 2 and Graph 2, indicating that none of these chemomechanical agents tested in the study have brought about any chemical alterations in the dentin surface.

## DISCUSSION

Bonding to caries-affected dentin is always considered very challenging. Besides presenting a significant reduction in its mineral content, caries-affected dentin undergoes an important loss in the crystallinity of its remaining mineral phase, as well as considerable changes in the secondary structure of its organic components. The penetration of resin monomers into the dentin tubules of caries-affected dentin is usually impaired due to the presence of acid-resistant mineral casts, like those formed by b-tricalcium phosphates. Such structures, also called “whitlockite,” are deposited into the dentin tubules during the early phases of dentin reaction against further caries progression. Besides hindering intratubular penetration, such enhanced impermeability impairs also the sideways diffusion of resin monomers via the dentin tubules to the deepest demineralized dentin. This poorly infiltrated substrate, along with the low mechanical properties of the caries-affected dentin, has been pointed out as the most logical explanation for the lower bond strengths obtained to such a substrate as compared with that to sound dentin. The majority of laboratory studies evaluating the bond strength of adhesive systems have used normal dentin as a substrate, which does not simulate the actual clinical scenario.^14-16^ In this research work, carious molars in which caries extending beyond the middle third of dentin was selected with 1 to 1.5 mm of dentin remaining for the bonding and caries excavation was limited to the removal of outer highly infected and denatured dentin, preserving the inner layer of caries-affected dentin that still possesses a high potential to remineralize, in order to simulate a clinical scenario. In order to eliminate the substrate-related variation in the bonding of composite resin, the same teeth were sectioned longitudinally through the center of the carious lesion in a buccolingual direction into two halves.

**Table 1:** **Table 1:** Microtensile bond strength values

								*95% CI for mean*							
*Group*		*Mean*		*Standard deviation*		*SEM*		*Lower bound*		*Upper bound*		*Min*		*Max*	
Control		20.301		3.500		0.904		18.363		22.239		15.550		27.150	
Carisolv		21.454		2.242		0.579		20.213		22.695		18.310		25.300	
Papacarie		22.001		3.85		0.74		19.22		25.55		17.95		28.50	
Carie-care		21.548		2.638		0.681		20.087		23.009		18.420		25.900	

pSEM: Standard error of mean; CI: Confidence interval

**Table 2:** **Table 2:** Energy-dispersive X-ray values

*Group*		*Mean*		*Standard deviation*	
Control		2.173		0.166	
Carisolv		2.087		0.067	
Papacarie		2.078		0.075	
Carie-care		2.096		0.062	

Mean Ca/P ratio of four groups tested in the study with a p-value of 0.296; No statistically significant difference among all the four groups

The self-limiting action of Carisolv is due to col-lagenolytic activity of the sodium hypochlorite present in it which affects only infected dentin and leaves intact caries-affected dentin. The dentinal surfaces formed after biochemical caries removal are very irregular with many overhangs and undercuts. In addition, the biochemical method removes the smear layer completely and exposes dentinal tubules.^12,17^

Papacarie, a gel based on papain, chloramines, and toludine blue, has similar use, indication, and chemomechanical caries removal efficacy as Carisolv. It is more palatable and has marginal reduction in time consumption than Carisolv. Papain similar to human pepsin makes it easy to clean necrotic tissues and secretions and reduces tissue repair time in addition to not affecting sound tissues close to lesion. It does not harm healthy tissue and accelerates tissue healing. It acts only on carious tissue, which lacks the plasmatic protease inhibitor alpha-1-antitrypsin; its proteolytic action is inhibited on healthy tissue, which contains this substance. In addition to papain, the chloramines present in the product have the potential of dissolving carious dentin by means of chlori-nation of the partially degraded collagen. This mechanism affects the collagen structure, dissolving hydrogen bonds and thus facilitating tissue removal in the same way as Carisolv.^9-11^ Carie-care gel, also based on papain, works on the same principle as that of Papacarie, Sano et al^18^ introduced microtensile testing to dentistry to measure the ultimate tensile strength and modulus of elasticity of mineralized and demineralized dentin. Advantages for the microtensile bond strength approach include fewer cohesive failures due to better stress distribution during loading, measurement of higher interfacial bond strengths, permitting testing of irregular surfaces, very small areas, and facilitating scanning electron microscope/transmission electron microscope examinations of the failed bonds since the surface area is approximately 1 mm^2^. In this study dumbbell-shaped specimens are used which are smallest at the adhesive interface and stresses are directed to it so that fracture of the specimens initiates at the weakest region of the tested interface. This method eliminates most of the cohesive resin or dentin fractures seen in more traditional tensile strength test procedures that are due to nonuniform stress distributions.^19^

On analyzing the results of the study it was observed that the bond strength values recorded for all groups tested (control and experimental groups) ranged between 15.5 and 27.2 MPa as against the bond strength values obtained in many other studies where values as high as 33.1 ± 3.8 MPa have been recorded. That may be because most of the studies conducted are on carious dentin in the occlusal and middle third of the dentin.^16,20^ Superficial dentin normally results in higher bond strength than deep dentin. The water content of dentin near the dentinoenamel junction is about 1% (volume), while that of dentin near pulp is about 22%. This difference in intrinsic moisture has been deemed responsible for the difference in bond strength between superficial and deep dentin. Suzuki and Finger^21^ reported that bond strength decreased 30 to 40% in deep dentin for three adhesives, whereas Nakamichi et al^22^ reported a 50% decrease in bond strength from superficial to deep dentin in bovine teeth. In our study, the samples selected were with caries extending up to 1 to 1.5 mm from pulp. Therefore, the low bond strength values were on account of altered dentin surface rather than the action of chemomechanical caries removal *per se.*

On comparing the microtensile bond strength of various groups tested in this study, it was found that there is no statistically significant difference between the control and the experimental groups. However, a marginal increase in bond strength values of chemomechani-cal agents group was observed as compared with bur group. Caries removal with these agents does not produce smear layer, resulting in greater opening of the dentinal tubules, which optimizes the penetration of the adhesive systems. These results were in accordance with the study done by Aggarwal et al^20^ where they have evaluated the influence of Carisolv and conventional caries excavation on the microtensile bond strength of three different adhesive systems and have concluded that there was no statistically significant effect of different caries excavation method on bond strength values, whereas the etch and rinse adhesive and two bottle self-etch system showed significantly higher bond strength than the single bottle self-etch system with no difference between the former two adhesive systems.

An elemental analysis of dentin surface after treatment with these chemomechanical agents is done using a noninvasive EDX system that is based on the generation of characteristic X-rays in atoms of the specimen by the incident beam electron. This technique is nondestructive; specimen of interest can be examined with little or no sample preparation and also facilitates multielement detection.^23^ This has been used to investigate the calcium (Ca) and phosphorous (P) content of dental hard tissue in several studies as well as delineate variations within dental hard tissues.^24,25^

In the current study, EDX results of the control bur group and three chemomechanical agents were in the same range reported in a study by Hamama et al.^26^ Hence, the mineral content of dentin was unaffected by chemomechanical caries removal agents as demonstrated in previous studies.^27,28^

To summarize the data analysis, none of the che-momechanical methods had any influence on the bond strength of the adhesive system to carious dentin. This indicates that none of the products seem to alter the dental substrate, which could interfere in the bond strength values. One of the major shortcomings of Carisolv and Papacarie is that the product is expensive. Caricare being a papain-based chemomechanical caries removal agent, which is indigenously manufactured in our country, costs much less than Carisolv and Papacarie and hence can be considered as an equally effective economical alternative.

## CLINICAL SIGNIFICANCE

- Chemomechanical agents are alternative to conventional method where use of local anesthesia and burs is avoided, which make pediatric patients more cooperative to dental treatment.
- Carie-care, a newer papain-based product, is an economic alternative to other chemomechanical agents like Papacarie and Carisolv.
- Pulp capping procedures are of great value in deep-seated carious lesions, especially pediatric cases, as it preserves the vitality of young developing tooth.
